# An Excess Electron Bound to Magnesium Halides and Basic Grignard Compounds (RMgX and RMgR, R = Me, Et, Ph; X = F, Cl, Br)

**DOI:** 10.1021/acs.jpca.1c00750

**Published:** 2021-03-10

**Authors:** Jakub Brzeski, Sylwia Freza, Marcin Czapla, Piotr Skurski

**Affiliations:** † Laboratory of Quantum Chemistry, Faculty of Chemistry, University of Gdańsk, Wita Stwosza 63, 80-308 Gdańsk, Poland; ‡ Henry Eyring Center for Theoretical Chemistry, Department of Chemistry, University of Utah, Salt Lake City, Utah 84112, United States

## Abstract

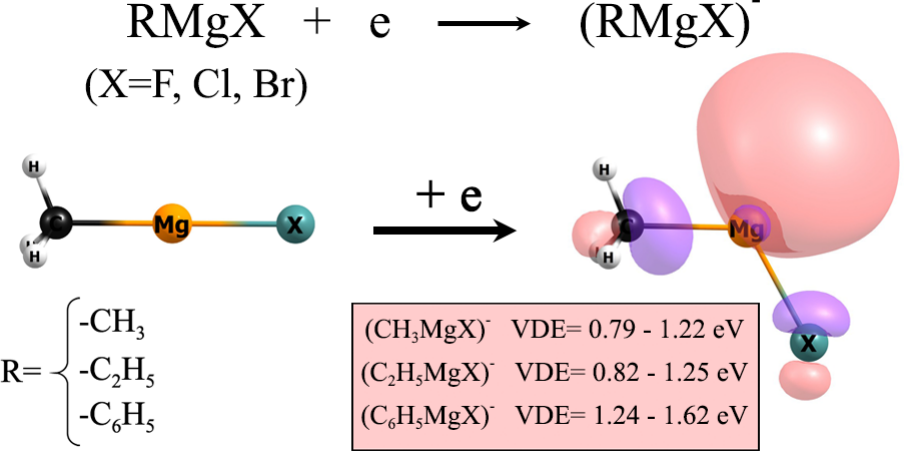

Grignard reagents are commonly used in organic synthesis, yet their ability to form stable anionic states has not been recognized thus far. In this work, representative examples of RMgF, RMgCl, and RMgBr molecules involving methyl, ethyl, and phenyl functional groups serving as R substituents are investigated regarding their equilibrium structures, adiabatic electron affinities, and vertical electron detachment energies of their daughter anions. The electronic stabilities determined for the negatively charged Grignard compounds are then compared to those predicted for their corresponding magnesium halides. The anions formed by RMgX (R = Me, Et, Ph; X = F, Cl, Br) molecules are found to be adiabatically electronically stable valence-bound systems characterized by relatively large vertical electron detachment energies spanning the 0.79–1.62 eV range. In addition, significant structural relaxation upon attachment of an excess electron is predicted for all Grignard compounds considered. Furthermore, the re-examination of the anions formed by magnesium halides resulted in recognizing them as valence-bound rather than dipole-bound anions, in contrast to the earlier interpretations.

## 1 Introduction

Since the discovery and isolation of organomagnesium compounds (later termed Grignard reagents, GRs) in 1900 by Nobel Prize-winning French chemist François Auguste Victor Grignard,^1^ compounds with the generic formula R–Mg–X (where X is a halogen and R is an organic group, typically an alkyl or aryl) have been commonly utilized in organic synthesis to create new carbon–carbon bonds (e.g., during the alkylation of aldehydes and ketones) and for the formation of carbon–phosphorus, carbon–tin, carbon–silicon, carbon–boron, and other carbon–heteroatom bonds.^2−7^ Grignard reagents are usually produced from the heated combination of organic halides and magnesium metal in the presence of either diethyl ether or tetrahydrofuran (required to stabilize the organomagnesium compound).^8,9^ Even though most GRs are stable in ethereal solution, oxygen and water coming from atmospheric moisture should be excluded (to prevent destroying the reagent by protonolysis or oxidation) using ultrasound and air-free techniques.^10,11^

Albeit Grignard compounds have been thoroughly investigated in the past, their ability to form stable anionic states has not been verified. Since the adiabatic electron affinity (EA) of a neutral molecule and the vertical electron detachment energy (VDE) of its daughter anion are very important features characterizing a given molecular system, it seems surprising that these properties are not yet established for such commonly used species as Grignard reagents. In fact, the importance and utility of EAs and VDEs extend beyond the regime of gas-phase ion chemistry as the properties of negative ions play a role in many areas of semiconductor chemistry,^12−16^ polymer photoluminescence,^17−21^ microelectronics,^22,23^ silicon chemistry,^24,25^ fullerene chemistry,^26−29^ and silicon quantum dot design.^30^ Since we are unaware of any studies on the negatively charged Grignard compounds, in this contribution we describe our efforts to verify the possibility of forming stable anionic states based on GR molecules and to evaluate their excess electron binding energies.

Despite the absence of any reports related to the excess electron binding to Grignard reagents, one may anticipate the stability of the (RMgX)^−^ anions by recalling the electronic stability of the (MgF_2_)^−^ system.^31,32^ Namely, the GR might be viewed as the magnesium halides having one halogen atom replaced with organic functional group R, hence the ability to bind an excess electron by RMgX systems could be weakened yet preserved to some extent (in the sense that the EAs of Grignard molecules could be expected to remain positive although likely smaller than those of magnesium halides). Unfortunately, literature reports describing the possibility of an excess electron binding by MgF_2_, MgCl_2_, MgBr_2_, and MgI_2_ are either scarce or lacking. Interestingly, the calculations performed 40 years ago by Seiders et al.^31^ resulted in estimating the adiabatic EA of MgF_2_ with decent accuracy (0.53 eV) despite the rather modest theoretical approach used (ΔSCF level). Moreover, the same research group properly anticipated that the inclusion of electron correlation would increase the EA by 0.05–0.20 eV.^31^ Indeed, as we will later demonstrate, the reliable theoretical estimation of the adiabatic electron affinity of MgF_2_ (0.661 eV) falls within the predicted 0.58–0.73 eV range. Although Seiders and co-workers claimed that the dipole potential of the bent neutral MgF_2_ molecule plays a crucial role in the binding of the excess electron,^31^ we do not consider the (MgF_2_)^−^ to be a dipole-bound anion. (Later we will have more to say about this issue when we illustrate the singly occupied molecular orbitals of the (MgF_2_)^−^, (MgCl_2_)^−^, and (MgBr_2_)^−^ anions and discuss their VDEs, as well as the EAs, VAEs (vertical electron attachment energies), and dipole moments of their corresponding neutral parents.)

In this work, we first describe our findings concerning the anions formed by magnesium halides, and then we move on to characterize the structures of the neutral and anionic Grignard systems utilizing methyl, ethyl, and phenyl as the functional group as well as the adiabatic electron affinities and vertical electron detachment energies of those species. In addition, we comment on the nature of the negatively charged states supported by MgX_2_, RMgX, and RMgR molecules (X = F, Cl, Br; R = CH_3_, C_2_H_5_, C_6_H_5_).

## 2 Methods

The stationary-point structures of MgX_2_ (X = F, Cl, Br), RMgX (X = F, Cl, Br; R = Me, Et, Ph), and RMgR (R = Me, Et) systems and their corresponding anions were obtained by applying the quadratic configuration interaction method with single and double substitutions (QCISD)^33−35^ with the aug-cc-pVDZ valence basis set.^36^ The harmonic vibrational frequencies characterizing the stationary points were evaluated at the same QCISD/aug-cc-pVDZ theory level to ensure that all of the obtained structures correspond to true minima or first-order saddle points on the potential energy surface.

The vertical electron detachment energies of the anions as well as the adiabatic electron affinities (not including zero-point vibrational corrections) and the vertical electron attachment energies of the neutral species were calculated by employing the indirect approach (i.e., by subtracting the energy of the anion from that of the neutral) involving the QCISD/aug-cc-pVDZ energies and by applying the outer valence Green function OVGF method (*B* approximation)^37−45^ together with the aug-cc-pVDZ basis sets. Because of the fact that the OVGF approximation remains valid only for outer valence ionization for which the pole strengths (PS) are greater than 0.80–0.85,^46^ we verified that the PS values obtained were sufficiently large to justify the use of the OVGF method.

Because of the limited computer resources available, the equilibrium structures of the neutral and negatively charged C_6_H_5_MgC_6_H_5_ systems were calculated by applying the second-order Møller–Plesset perturbation method (MP2)^47−49^ with the aug-cc-pVDZ basis set. The vertical electron detachment energy of the (C_6_H_5_MgC_6_H_5_)^−^ anion and the adiabatic electron affinity and the vertical electron attachment energy of the neutral C_6_H_5_MgC_6_H_5_ system were evaluated (using the indirect approach) by employing the MP2 method with the aug-cc-pVDZ basis set. In order to verify whether such obtained vertical electron detachment energy and adiabatic electron affinity are reliable, we performed the additional MP2/aug-cc-pVDZ calculations of the VDEs and EAs characterizing C_6_H_5_MgX neutral molecules and (C_6_H_5_MgX)^−^ anions (X = F, Cl, Br) (which we earlier estimated at the more advanced QCISD/aug-cc-pVDZ theory level). Since the EAs and VDEs calculated using the MP2 method with the aug-cc-pVDZ basis set were found to be only slightly smaller (by 0.01–0.03 eV) than those predicted at the QCISD/aug-cc-pVDZ level, we are confident that our MP2/aug-cc-pVDZ results presented here for the C_6_H_5_MgC_6_H_5_ and (C_6_H_5_MgC_6_H_5_)^−^ species can be considered to be reliable.

All calculations were carried out using the *Gaussian 16* (Rev. B.01) package.^50^

## 3 Results

To provide a clear description of the anions formed by neutral magnesium halides (MgX_2_, X = F, Cl, Br) and basic Grignard compounds (RMgX, R = Me, Et, Ph; X = F, Cl, Br), we organized our discussion into four subsections, each of which contains the results (including tables and figures) related to a certain group of compounds. While discussing the vertical electron detachment energies of the anions studied, we focus on the values obtained by employing the QCISD method (which was also used to determine the equilibrium geometries) whereas the VDEs calculated using the OVGF method are provided for comparison only. The Cartesian coordinates of all neutral and anionic systems investigated in this work are provided in the Supporting Information (Tables S1–S4).

### 3.1 MgX_2_ and (MgX_2_)^−^ Systems (X = F, Cl, Br)

**Figure 1:**
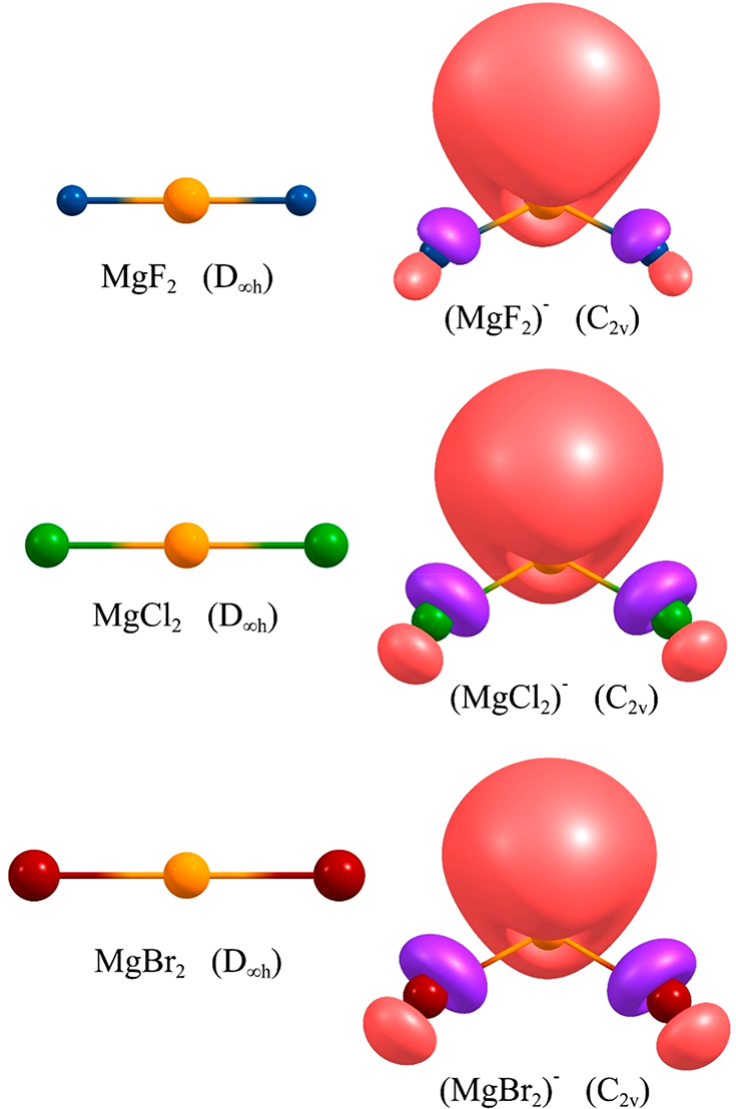
Structures of MgX_2_ (X = F, Cl, Br) neutral systems (left) and their daughter anions (right). Singly occupied molecular orbitals depicted for the anions are plotted with a contour value of 0.03 e/Å^3^.

Equilibrium structures of the neutral MgX_2_ (X = F, Cl, Br) molecules and their corresponding (MgX_2_)^−^ anions are shown in Figure 1, whereas the Cartesian coordinates are provided in the Supporting Information (Table S1). As recognized a few decades ago both experimentally (via electric deflection and mass spectrometric detection^51,52^ and by performing electron diffraction measurements^53,54^) and theoretically (by nonempirical studies),^31,55^ neutral alkaline earth dihalides containing either Be or Mg as a central atom adopt linear *D*_∞*h*_-symmetry geometries. Indeed, our ab initio calculations revealed that MgF_2_, MgCl_2_, and MgBr_2_ are linear closed-shell systems having vanishing dipole moment and considerable quadrupole moments. The predicted Mg–X bond lengths of 1.768 Å (in MgF_2_), 2.210 Å (in MgCl_2_), and 2.356 Å (in MgBr_2_) reflect the effective ionic radii of X substituents (1.33, 1.81, and 1.96 Å for fluorine, chlorine, and bromine, respectively).^56^ Despite the absence of bound virtual orbitals in the neutral magnesium halides, we found the vertical electron attachment energies (VAE) of the MgF_2_, MgCl_2_, and MgBr_2_ systems to be positive (0.23–0.49 eV, see Table 1). In particular, our VAE estimate for MgF_2_ (0.23 eV) agrees well with the theoretical prediction reported by the Sommerfeld group (0.269 eV).^32^

**Table 1:** Adiabatic Electron Affinities (EA in eV), Vertical Electron Detachment Energies (VDE in eV), and Vertical Electron Attachment Energies (VAE in eV) of the MgX_2_/(MgX_2_)^−^ (X = F, Cl, Br) Systems Calculated at the QCISD/aug-cc-pVDZ Level^a^

system	⟨*S*^2^⟩	symmetryneutral/anion	dipole moment	EA	VDE	VAE
MgF_2_/(MgF_2_)^−^	0.7504	*D*_∞*h*_/*C*_2*v*_	μ^N^ = 0.00, μ^A^ = 6.45	0.66	1.29(1.34)	0.23
MgCl_2_/(MgCl_2_)^−^	0.7501	*D*_∞*h*_/*C*_2*v*_	μ^N^ = 0.00, μ^A^ = 6.67	1.07	1.87(1.94)	0.43
MgBr_2_/(MgBr_2_)^−^	0.7514	*D*_∞*h*_/*C*_2*v*_	μ^N^ = 0.00, μ^A^ = 6.41	1.17	1.97(2.05)	0.49

a Dipole moments μ^N^ and μ^A^ (in Debye) are determined for the neutral systems at the equilibrium structure of the neutral and anion, respectively. The VDEs given in parentheses correspond to the values obtained at the OVGF/aug-cc-pVDZ level. The expectation values of the *S*^2^ operator in the UHF wavefunctions that accompany the OVGF calculations of VDE are provided as ⟨*S*^2^⟩.

Although the positive VAEs could indicate the possibility of forming geometrically stable linear (MgX_2_)^−^ anions, we verified that the ^2^Σ_g_ (MgX_2_)^−^*D*_∞*h*_-symmetry anions do not correspond to local minima on the anionic potential energy surface. Instead, the presence of low-energy degenerate π_u_ virtual orbitals in the *D*_∞*h*_-symmetry MgX_2_ neutral systems causes a first-order Jahn–Teller instability when an excess electron is attached, which leads to bent structures of the (MgX_2_)^−^ anions (Figure 1). Our calculations revealed that (MgF_2_)^−^, (MgCl_2_)^−^, and (MgBr_2_)^−^ anions adopt significantly bent equilibrium structures with *C*_2*v*_-symmetry with X–Mg–X valence angles of ca. 118–124° and the Mg–X bond lengths slightly longer (by 0.07–0.17 Å) than those predicted for the corresponding neutral compounds. The fully symmetrical a_1_ singly occupied molecular orbitals (SOMO) of the (MgX_2_)^−^ anions are localized primarily on the Mg atom, although the contributions on the halide atoms are also substantial (Figure 1). Both the adiabatic electron affinities spanning the 0.66–1.17 eV range and the vertical electron detachment energies spanning the 1.29–1.97 eV range (Table 1) indicate that the (MgX_2_)^−^ systems represent relatively strongly bound molecular anions whose electronic stability is comparable to many valence-bound negatively charged species.^57−60^ The nature of the (MgX_2_)^−^ anions (i.e., identification of the potential primarily responsible for an excess electron binding) seems to be a particularly interesting issue as their neutral parents exhibit substantial dipole moments at the bent anionic structures which could indicate the dipole-bound character of the anionic states. In fact, such an interpretation was proposed in the late 1970s by Seiders and co-workers for the (MgF_2_)^−^ anion, whose electronic stability was recognized as resulting from the dipole potential of the bent neutral MgF_2_ molecule.^31^ However, we do not share this point of view, as we now explain.

Although it is well established that the excess electron binding energies of dipole-bound anions (DBS) depend on the dipole moments of their parent neutral molecules,^61−63^ they remain relatively small values (i.e., rarely exceeding 0.3 eV)^64^ whereas the VDEs found for (MgX_2_)^−^ (X = F, Cl, Br) exceed 1 eV and approach 2 eV (for X = Cl, Br) (Table 1). In particular, the VDEs of dipole-bound anions based on the systems exhibiting dipole moments of their neutral parents similar to those of the bent MgX_2_ species (6.4–6.7 D, see Table 1) are significantly smaller than those of the (MgX_2_)^−^ (e.g., the VDE of 0.08 eV was predicted for (C_5_H_2_)^−^ (μ(C_5_H_2_) = 6.28 D)^62^ and the VDE of 0.21 eV was evaluated for ((HF)_3_)^−^ (μ((HF)_3_) = 6.54 D)^65^). Next, the spatial extent of the excess electron density in the (MgX_2_)^−^ systems is considerably smaller than the spatial extent usually characterizing typical dipole-bound anionic states (cf. the SOMO orbitals depicted in refs (61−64).). Unlike in dipole-bound anions where very diffuse basis functions are necessary to describe an excess electron, an unpaired electron in the (MgX_2_)^−^ anions can be described by the valence orbitals, similarly to typical valence-bound anions.^57,60^ The presence of substantial contributions from ligands’ atomic orbitals (AOs) to SOMO in the (MgX_2_)^−^ systems is yet another difference between them and DBS anions as the SOMO in the latter species is always very diffuse and localized outside the molecular framework.^62−65^ In addition, if the electronic stability of the (MgX_2_)^−^ species were related primarily to the interaction of an excess electron with a molecular dipole, then the VDE of (MgCl_2_)^−^ would exceed the VDE of (MgBr_2_)^−^ because the dipole moment of MgCl_2_ (determined for its anionic bent structure) is larger by 0.26 D than the dipole moment of MgBr_2_ (determined for the bent structure of (MgBr_2_)^−^), whereas our calculations indicate the exact opposite (i.e., the VDE of (MgBr_2_)^−^ is larger by 0.1 eV than the VDE of (MgCl_2_)^−^, see Table 1). Therefore, we conclude that the (MgF_2_)^−^, (MgCl_2_)^−^, and (MgBr_2_)^−^ systems, although showing a resemblance to dipole-bound anions, should be considered to be valence-bound negatively charged species due to their large excess electron binding energies and small spatial extent of excess electron density (indicating the key role of the valence molecular orbitals).

### 3.2 CH_3_MgX (X = F, Cl, Br) and CH_3_MgCH_3_ Systems and Their Corresponding Anions

**Figure 2:**
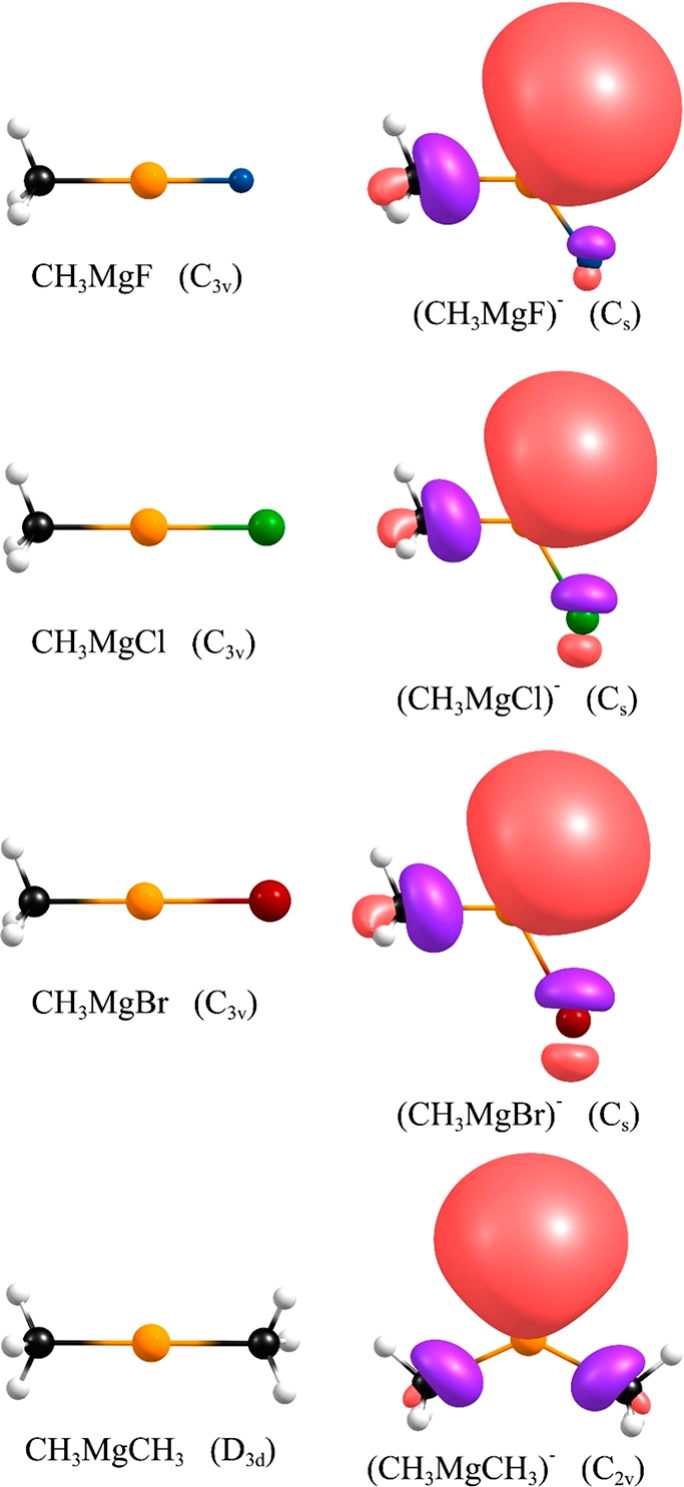
Structures of the CH_3_MgX (X = F, Cl, Br) and CH_3_MgCH_3_ neutral systems (left) and their daughter anions (right). Singly occupied molecular orbitals depicted for the anions are plotted with a contour value of 0.03 e/Å^3^.

Having discussed the possibility of an excess electron binding to magnesium halides, we now move on to describing the anions formed by basic Grignard compounds. Since the simplest systems of that type match the CH_3_MgX formula (i.e., RMgX with the methyl group serving as R), we began our investigation from examining the structures and properties of such molecules. The equilibrium geometries of the neutral CH_3_MgX systems (X = F, Cl, Br) are shown in Figure 2, whereas the Cartesian coordinates are provided in the Supporting Information (Table S2).

The CH_3_MgX neutral molecules (X = F, Cl, Br) adopt *C*_3*v*_-symmetry equilibrium structures with the C–Mg–X linear fragments. As indicated by their dipole moments (1.88–2.22 D, see the μ^N^ values in Table 2), CH_3_MgX systems are polar (with their dipole moment vector along the *C*_3_ symmetry axis) yet not polar enough to support the existence of dipole-bound anionic states.^64^ Despite this, we found the VAE values to be positive (although rather small) for CH_3_MgCl and CH_3_MgBr molecules, whereas negative VAE was predicted for CH_3_MgF (Table 2).

**Table 2:** Adiabatic Electron Affinities (EA in eV), Vertical Electron Detachment Energies (VDE in eV), and Vertical Electron Attachment Energies (VAE in eV) of the CH_3_MgX/(CH_3_MgX)^−^ (X = F, Cl, Br) and CH_3_MgCH_3_/(CH_3_MgCH_3_)^−^ Systems Calculated at the QCISD/aug-cc-pVDZ Level^a^

system	⟨*S*^2^⟩	symmetry neutral/anion	dipole moment	EA	VDE	VAE
CH_3_MgF/(CH_3_MgF)^−^	0.7519	*C*_3*v*_/*C_s_*	μ^N^ = 1.88, μ^A^ = 5.13	0.26	0.79(0.85)	<0
CH_3_MgCl/(CH_3_MgCl)^−^	0.7524	*C*_3*v*_/*C_s_*	μ^N^ = 2.22, μ^A^ = 5.86	0.47	1.13(1.20)	0.02
CH_3_MgBr/(CH_3_MgBr)^−^	0.7526	*C*_3*v*_/*C_s_*	μ^N^ = 2.13, μ^A^ = 6.01	0.54	1.22(1.30)	0.05
CH_3_MgCH_3_/(CH_3_MgCH_3_)^−^	0.7516	*D*_3*d*_/*C*_2*v*_	μ^N^ = 0.00, μ^A^ = 3.70	0.01	0.46(0.50)	<0

a Dipole moments μ^N^ and μ^A^ (in Debye) are determined for the neutral systems at the equilibrium structure of the neutral and anion, respectively. The VDEs given in parentheses correspond to the values obtained at the OVGF/aug-cc-pVDZ level. The expectation values of the *S*^2^ operator in the UHF wavefunctions that accompany the OVGF calculations of VDE are provided as ⟨*S*^2^⟩.

An excess electron attachment to any of the CH_3_MgX (X = F, Cl, Br) molecules leads to a first-order Jahn–Teller distortion (due to the presence of low-energy degenerate e-symmetry virtual orbitals in the neutral systems), which results in adopting bent *C_s_*-symmetry equilibrium structures of the corresponding (CH_3_MgX)^−^ anions with the C–Mg–X valence angles spanning the 118.23–124.75° range. (See Figure 2 and also Table S2 in the Supporting Information.) The adiabatic electron affinities of the CH_3_MgX (X = F, Cl, Br) compounds are positive yet smaller by ca. 0.4–0.6 eV than those of the corresponding MgX_2_ (X = F, Cl, Br) species (cf. Tables 1 and 2), which is likely caused by the presence of only one electronegative substituent X in the former systems. Still, the (CH_3_MgX)^−^ species remain relatively strongly bound anions as their VDEs span the 0.79–1.22 eV range. Like the EA values, the VDE values determined for the (CH_3_MgX)^−^ anions are smaller (by 0.5–0.7 eV) than those of their corresponding (MgX_2_)^−^ anions (cf. Tables 1 and 2). Keeping in mind our discussion concerning the nature of the negatively charged (MgX_2_)^−^ states (see the preceding section), we consider the (CH_3_MgX)^−^ systems to be valence-bound anions because (i) their VDE values are large (approaching or exceeding 1 eV) and thus cannot result from the stabilizing interaction of the excess electron and the dipole moment of the bent neutral molecule (μ^A^ = 5.13–6.01 D, see Table 2), (ii) the spatial extent of the excess electron density is rather small, which indicates the key role of the valence molecular orbitals, and (iii) the distribution of the excess electron density (manifested here by the localization and shape of the SOMO orbital) is not consistent with the dipole potential exhibited by the neutral molecular framework because the positive pole of a molecular dipole is not localized near the Mg atom but in between the Mg atom and the methyl group. Clearly, the unpaired electron is assigned to a Mg hybrid orbital having antibonding relationships with the X and CH_3_ ligands, which confirms the valence rather than the diffuse, nonvalence (e.g., dipole) character of the orbital capturing an excess electron.

For reasons of completeness, we also examined the possibility of forming a stable anionic state by the CH_3_MgCH_3_ molecule (despite the fact that this system does not represent a Grignard compound). Since we found that replacing one halogen atom in MgX_2_ with the methyl group decreases both the adiabatic and vertical excess electron binding energy, we decided to verify whether the replacement of two halogen atoms in MgX_2_ with CH_3_ would render the resulting anion electronically unstable. The equilibrium structure of the neutral CH_3_MgCH_3_ molecule was found to correspond to the *D*_3*d*_ symmetry configuration (see Figure 2 and Table S2 in the Supporting Information) with no net dipole moment. Although the EA of this system is very small (0.006 eV), it remains positive, which indicates the adiabatic electronic stability of its daughter anion. Similarly to (MgX_2_)^−^ and (CH_3_MgX)^−^ systems, the structure of the (CH_3_MgCH_3_)^−^ anion is significantly bent. (The C–Mg–C valence angle was determined to be equal to 127.82°). According to our calculations, the VDE of the *C*_2*v*_-symmetry (CH_3_MgCH_3_)^−^ anion approaches 0.5 eV (Table 2), which means that this system preserves its ability to form a stable anionic state even in the absence of any electronegative substituents. Finally, we stress that (CH_3_MgCH_3_)^−^ is yet another system we consider to be a valence-bound rather than a dipole-bound anion (for the reasons emphasized in the preceding section and also due to the fact that the 3.7 D dipole moment (see the μ^A^ value in Table 2) characterizing the bent *C*_2*v*_-symmetry CH_3_MgCH_3_ molecular framework could possibly be responsible only for very weak excess electron binding of ca. 0.01–0.06 eV,^63,64^ whereas the vertical electronic stability of the (CH_3_MgCH_3_)^−^ anion is larger by an order of magnitude).

### 3.3 C_2_H_5_MgX (X = F, Cl, Br) and C_2_H_5_MgC_2_H_5_ Systems and Their Corresponding Anions

**Figure 3:**
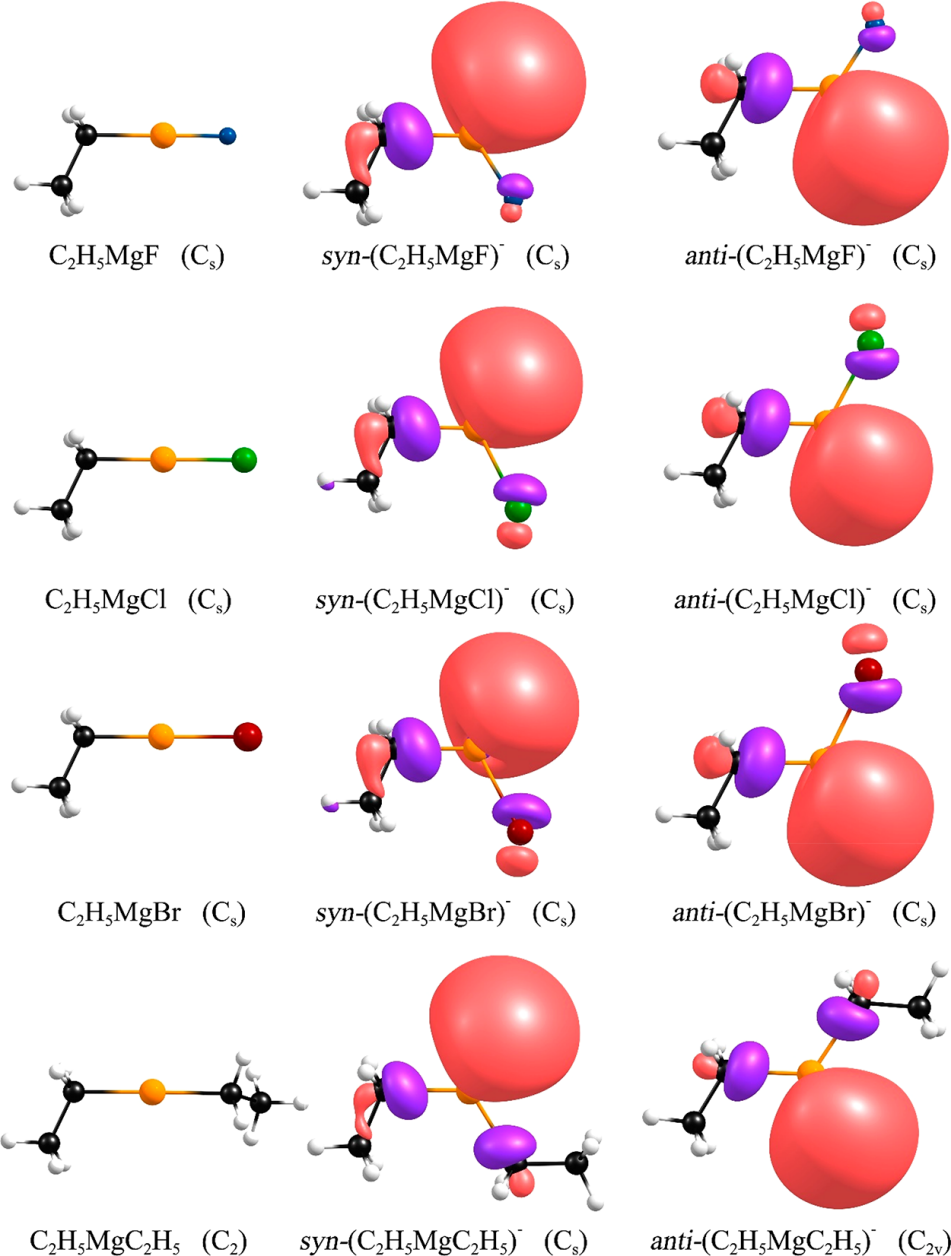
Structures of the C_2_H_5_MgX (X = F, Cl, Br) and C_2_H_5_MgC_2_H_5_ neutral systems (left) and their daughter anions (center and right). For each anionic case, two nearly energetically degenerate isomers (syn and anti) are shown. Singly occupied molecular orbitals depicted for the anions are plotted with a contour value of 0.03 e/Å^3^.

The next group of Grignard compounds we examined consists of the species matching the C_2_H_5_MgX (X = F, Cl, Br) formula. Equilibrium structures of such neutral and anionic systems are shown in Figure 3, whereas the Cartesian coordinates are provided in the Supporting Information (Table S3). The neutral C_2_H_5_MgX molecules (X = F, Cl, Br) adopt *C_s_*-symmetry equilibrium structures with the linear C–Mg–X fragment and the C–C–Mg valence angles spanning the 115.68–115.86° range. Alike the CH_3_MgX systems, C_2_H_5_MgX molecules are not polar enough to support dipole-bound anionic states because their dipole moments do not exceed 2.39 D. (See the μ^N^ values in Table 3).

**Table 3:** Adiabatic Electron Affinities (EA in eV), Vertical Electron Detachment Energies (VDE in eV), and Vertical Electron Attachment Energies (VAE in eV) of the C_2_H_5_MgX/(C_2_H_5_MgX)^−^ (X = F, Cl, Br) and C_2_H_5_MgC_2_H_5_/(C_2_H_5_MgC_2_H_5_)^−^ Systems Calculated at the QCISD/aug-cc-pVDZ Level^a^

system	⟨*S*^2^⟩	symmetryneutral/anion	dipole moment	EA	VDE	VAE
C_2_H_5_MgF/*anti*-(C_2_H_5_MgF)^−^, Δ*E* = 0.00	0.7522	*C_s_*/*C_s_*	μ^N^ = 2.04, μ^A^ = 4.97	0.30	0.83(0.90)	<0
C_2_H_5_MgF/*syn*-(C_2_H_5_MgF)^−^, Δ*E* = 0.06	0.7522	*C_s_*/*C_s_*	μ^N^ = 2.04, μ^A^ = 5.23		0.82(0.90)	
C_2_H_5_MgCl/*syn*-(C_2_H_5_MgCl)^−^, Δ*E* = 0.00	0.7527	*C_s_*/*C_s_*	μ^N^ = 2.39, μ^A^ = 5.96	0.51	1.15(1.24)	<0
C_2_H_5_MgCl/*anti*-(C_2_H_5_MgCl)^−^, Δ*E* = 0.03	0.7527	*C_s_*/*C_s_*	μ^N^ = 2.39, μ^A^ = 5.72		1.15(1.24)	
C_2_H_5_MgBr/*syn*-(C_2_H_5_MgBr)^−^, Δ*E* = 0.00	0.7530	*C_s_*/*C_s_*	μ^N^ = 2.30, μ^A^ = 6.13	0.58	1.25(1.35)	0.02
C_2_H_5_MgBr/*anti*-(C_2_H_5_MgBr)^−^, Δ*E* = 0.12	0.7529	*C_s_*/*C_s_*	μ^N^ = 2.30, μ^A^ = 5.88		1.25(1.34)	
C_2_H_5_MgC_2_H_5_/*syn*-(C_2_H_5_MgC_2_H_5_)^−^, Δ*E* = 0.00	0.7519	*C*_2_/*C_s_*	μ^N^ = 0.34, μ^A^ = 3.93	0.10	0.59(0.65)	<0
C_2_H_5_MgC_2_H_5_/*anti*-(C_2_H_5_MgC_2_H_5_)^−^, Δ*E* = 0.13	0.7519	*C*_2_/*C*_2*v*_	μ^N^ = 0.34, μ^A^ = 3.50		0.58(0.64)	

a Dipole moments μ^N^ and μ^A^ (in Debye) are determined for the neutral systems at the equilibrium structure of the neutral and anion, respectively. The VDEs given in parentheses correspond to the values obtained at the OVGF/aug-cc-pVDZ level. Relative energies (*ΔE*) of syn and anti anionic conformers are given in kcal/mol. The expectation values of the *S*^2^ operator in the UHF wavefunctions that accompany the OVGF calculations of VDE are provided as ⟨*S*^2^⟩.

The structural relaxation of the C_2_H_5_MgX molecules upon attachment of an excess electron is significant because the equilibrium geometries of the corresponding (C_2_H_5_MgX)^−^ anions contain strongly bent CH_2_–Mg–X fragments (the C–Mg–X valence angles span the 114.24–118.26° range) (Figure 3). As revealed by our calculations, two conformational isomers can be formed in the case of each (C_2_H_5_MgX)^−^ anion. Since these isomers can be interconverted by rotation about a formally single C–Mg bond, we call them syn and anti conformers to distinguish between the CH_3_ and X moieties localized on the same side and on the opposing sides of the H_2_C–Mg bond, respectively (Figure 3). The relative energies (*ΔE*) collected in Table 3 are very small (0.03–0.12 kcal/mol , which indicates that the syn and anti conformers of each (C_2_H_5_MgX)^−^ anion are practically isoenergetic. To verify whether the syn and anti conformers may interconvert easily, we calculated the kinetic barrier heights for syn → anti isomerization as equal to 0.36 kcal/mol (for (C_2_H_5_MgF)^−^), 0.44 kcal/mol (for (C_2_H_5_MgCl)^−^), and 0.53 kcal/mol (for (C_2_H_5_MgBr)^−^). Clearly, these kinetic barriers are small enough to make the syn and anti conformers of each (C_2_H_5_MgX)^−^ anion fluxional near room temperatures.

As far as the excess electron binding energies of the (C_2_H_5_MgX)^−^ species are concerned, we found these systems to be adiabatically electronically stable, having EA values slightly larger (by 0.04 eV) than their corresponding (CH_3_MgX)^−^ compounds. In fact, the same trend can be seen when the vertical excess electron binding energies of (C_2_H_5_MgX)^−^ are compared to those of their corresponding (CH_3_MgX)^−^ systems. Namely, the VDEs of 0.83, 1.15, and 1.25 eV predicted for (C_2_H_5_MgF)^−^, (C_2_H_5_MgCl)^−^, and (C_2_H_5_MgBr)^−^, respectively, are larger by ca. 0.02–0.04 eV than those determined for the (CH_3_MgX)^−^ compounds containing the same halogen substituent (cf. Tables 2 and 3). Somewhat surprisingly, these differences in VDEs obtained for (C_2_H_5_MgX)^−^ and (CH_3_MgX)^−^ anions do not reflect the fact that the methyl group is a slightly better electron acceptor than the ethyl group (the electron affinity of CH_3_ is very small yet positive (0.08 ± 0.03 eV)^66^ whereas that of C_2_H_5_ is negative^67^). In addition, we found that the VDEs characterizing syn and anti conformers of any (C_2_H_5_MgX)^−^ anion examined are nearly the same, as the fifth column in Table 3 affirms.

**Figure 4:**
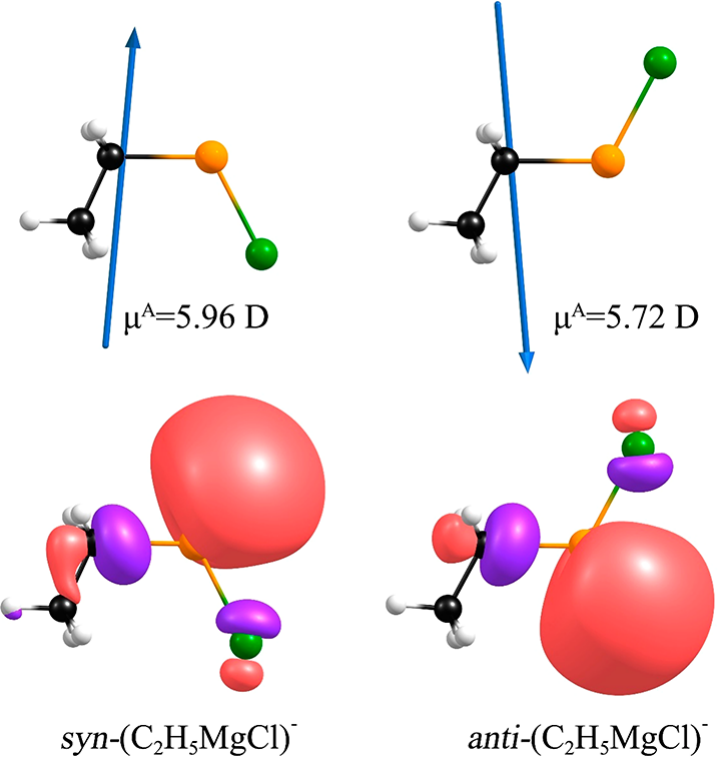
Dipole moments of the neutral C_2_H_5_MgCl molecule determined for the equilibrium structures of syn-(C_2_H_5_MgCl)^−^ (left) and anti-(C_2_H_5_MgCl)^−^ (right) anions and singly occupied molecular orbitals (plotted with the contour value of 0.03 e/Å^3^) for the syn-(C_2_H_5_MgCl)^−^ (left) and anti-(C_2_H_5_MgCl)^−^ (right) anions.

As mentioned above, the C_2_H_5_MgX molecules undergo substantial geometry relaxation upon excess electron attachment. Certainly, such a structural relaxation causes an increase in the dipole moment of the neutral molecular framework as indicated by the μ^A^ = 4.97–6.13 D values shown in Table 3. Nevertheless, we consider the (C_2_H_5_MgF)^−^, (C_2_H_5_MgCl)^−^, and (C_2_H_5_MgBr)^−^ systems to be valence-bound rather than dipole-bound anions for the reasons emphasized in the preceding sections (i.e., relatively large excess electron binding energies and small spatial extent of the excess electron density). Moreover, the distribution of SOMOs in the (C_2_H_5_MgX)^−^ anions (Figure 3) is not exactly consistent with the dipole potentials exhibited by the underlying neutral molecules as we verified that in the case of each system the positive pole of the molecular dipole is localized near the ethyl moiety rather than in the vicinity of the Mg atom. (See Figure 4, where two representative species are depicted with their SOMOs and dipole moment vectors.)

As in the case of the (CH_3_MgX)^−^ (X = F, Cl, Br) systems described in the preceding section, we decided to verify whether the (C_2_H_5_MgX)^−^ anions retain their electronic stability when the electronegative halogen atom, X, is replaced with the C_2_H_5_ alkyl group. We found that the neutral C_2_H_5_MgC_2_H_5_ system adopts a *C*_2_-symmetry equilibrium structure with the linear C–Mg–C fragment, which results in nearly vanishing polarity manifested by the dipole moment of 0.34 D (Figure 3 and Table 3). Excess electron attachment to the C_2_H_5_MgC_2_H_5_ molecule may lead to either the syn or anti isomer of the (C_2_H_5_MgC_2_H_5_)^−^ anion whose electronic energies differ by only 0.13 kcal/mol. Since we found the rotation about the C–Mg bond in (C_2_H_5_MgC_2_H_5_)^−^ to be essentially free (it requires surmounting a kinetic barrier of only 0.48 kcal/mol), it appears that the *syn*-(C_2_H_5_MgC_2_H_5_)^−^ and *anti*-(C_2_H_5_MgC_2_H_5_)^−^ conformers are fluxional near room temperature. The VDE values predicted for these anions are approximately the same (0.58–0.59 eV) and smaller than those of the (C_2_H_5_MgX)^−^ (X = F, Cl, Br) systems by 0.25–0.67 eV (Table 3). Hence, it turns out that replacing the halogen atom with the ethyl group in the C_2_H_5_MgX Grignard compounds does not render these molecules incapable of forming stable anions.

### 3.4 C_6_H_5_MgX (X = F, Cl, Br) and C_6_H_5_MgC_6_H_5_ Systems and Their Corresponding Anions

**Figure 5:**
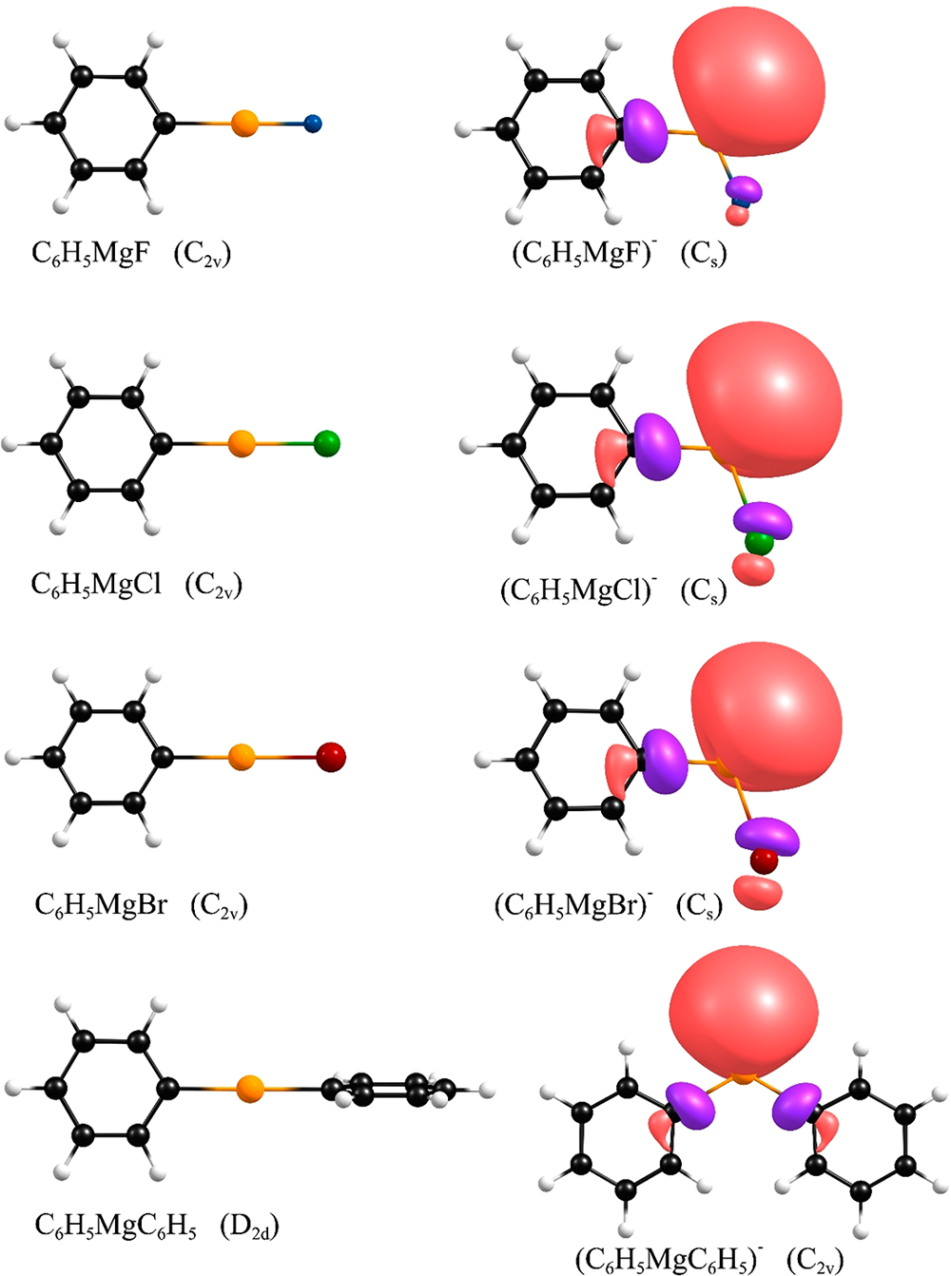
Structures of the C_6_H_5_MgX (X = F, Cl, Br) and C_6_H_5_MgC_6_H_5_ neutral systems (left) and their daughter anions (right). Singly occupied molecular orbitals depicted for the anions are plotted with a contour value of 0.03 e/Å^3^.

Having described the anions formed by attaching an excess electron to RMgX Grignard reagents containing alkyl functional groups (R = CH_3_, C_2_H_5_), we move on to presenting our findings concerning the C_6_H_5_MgX (X = F, Cl, Br) molecules which represent the simplest aromatic Grignard compounds. Equilibrium structures of such neutral and anionic systems are shown in Figure 5, whereas the Cartesian coordinates are provided in the Supporting Information (Table S4). The neutral C_6_H_5_MgX molecules (X = F, Cl, Br) adopt *C*_2*v*_-symmetry equilibrium structures whose dipole moments were found to span the 1.87–2.19 D range. (See the μ^N^ values in Table 4). Although the VAEs for these neutral structures are slightly positive (0.02–0.12 eV), we verified that the excess electron attachment to the C_6_H_5_MgX (X = F, Cl, Br) molecules leads to significantly bent structures with the C–Mg–X valence angles in the 111.50–116.66° range. The resulting *C_s_*-symmetry structures correspond to the global minima of the (C_6_H_5_MgX)^−^ (X = F, Cl, Br) anions (Figure 5).

**Table 4:** Adiabatic Electron Affinities (EA in eV), Vertical Electron Detachment Energies (VDE in eV), and Vertical Electron Attachment Energies (VAE in eV) of the C_6_H_5_MgX/(C_6_H_5_MgX)^−^ (X = F, Cl, Br) Systems Calculated at the QCISD/aug-cc-pVDZ Level and the C_6_H_5_MgC_6_H_5_/(C_6_H_5_MgC_6_H_5_)^−^ Systems Calculated at the MP2/aug-cc-pVDZ Level^a^

system	⟨*S*^2^⟩	symmetryneutral/anion	dipole moment	EA	VDE	VAE
C_6_H_5_MgF/(C_6_H_5_MgF)^−^	0.7517	*C*_2*v*_/*C_s_*	μ^N^ = 1.87, μ^A^ = 5.68	0.59	1.24(1.37)	0.02
C_6_H_5_MgCl/(C_6_H_5_MgCl)^−^	0.7520	*C*_2*v*_/*C*_s_**	μ^N^ = 2.19, μ^A^ = 6.14	0.80	1.54(1.68)	0.09
C_6_H_5_MgBr/(C_6_H_5_MgBr)^−^	0.7521	*C*_2*v*_/*C_s_*	μ^N^ = 2.12, μ^A^ = 6.22	0.86	1.62(1.76)	0.12
C_6_H_5_MgC_6_H_5_/(C_6_H_5_MgC_6_H_5_)^−^	0.7516	*D*_2*d*_/*C*_2*v*_	μ^N^ = 0.00, μ^A^ = 4.69	0.56	1.16(1.35)	<0

a Dipole moments μ^N^ and μ^A^ (in Debye) are determined for the neutral systems at the equilibrium structure of the neutral and anion, respectively. The VDEs given in parentheses correspond to the values obtained at the OVGF/aug-cc-pVDZ level. The expectation values of the *S*^2^ operator in the UHF wavefunctions that accompany the OVGF calculations of VDE are provided as ⟨*S*^2^⟩.

The adiabatic electron affinities predicted for the C_6_H_5_MgX (X = F, Cl, Br) molecules are systematically larger (by ca. 0.3 eV) than those obtained for the corresponding CH_3_MgX and C_2_H_5_MgX compounds but smaller than those calculated for the MgX_2_ systems (cf. Tables 1–4). We believe that this finding can be explained by the fact that the phenyl group is electronegative and may act as an electron acceptor, alike the halogen atoms. Since the EA of the C_6_H_5_ radical (1.096 ± 0.006 eV)^68^ is smaller than the EA of the fluorine (3.401 eV),^69^ chlorine (3.617 eV)^70^ or bromine (3.365 eV)^70^ atom, halogens appear to act as more effective electron-withdrawing substituents when embedded in a molecular system.^71^ The VDEs of the (C_6_H_5_MgX)^−^ (X = F, Cl, Br) anions were also predicted to be larger than those of the corresponding (RMgX)^−^ anions utilizing either a methyl or an ethyl group yet smaller than the VDEs of the (MgX_2_)^−^ systems (cf. Tables 1–4). Namely, we found VDE values of 1.24, 1.54, and 1.62 eV for (C_6_H_5_MgF)^−^, (C_6_H_5_MgCl)^−^, and (C_6_H_5_MgBr)^−^, respectively (Table 4). In the preceding sections, we explained the reasons that we consider the (MgX_2_)^−^ and (RMgX)^−^ (X = F, Cl, Br; R = CH_3_, C_2_H_5_) systems to be valence-bound rather than dipole-bound anions. In like fashion, we view the (C_6_H_5_MgX)^−^ species as valence-bound anions due to their large vertical excess electron binding energies (which cannot result from the dipole moments of their neutral parents) and relatively compact excess electron density distribution (as manifested by the SOMOs depicted in Figure 5).

Finally, we verified whether the (C_6_H_5_MgX)^−^ anions preserve their electronic stability when the electronegative halogen atom, X, is replaced with the second phenyl group. According to our calculations, the neutral C_6_H_5_MgC_6_H_5_ system adopts a *D*_2*d*_-symmetry equilibrium structure with the linear C–Mg–C fragment and two mutually perpendicular phenyl rings and thus a vanishing dipole moment (Figure 5). The positive EA value (0.56 eV) reflects the stability of the corresponding (C_6_H_5_MgC_6_H_5_)^−^ anion (Table 4). Alike for all of the previously discussed molecules, the geometry relaxation upon excess electron attachment to the C_6_H_5_MgC_6_H_5_ system is substantial as the (C_6_H_5_MgC_6_H_5_)^−^ anion adopts a planar *C*_2*v*_-symmetry equilibrium structure with a C–Mg–C valence angle of 119.51° (Figure 5). The VDE of 1.16 eV determined for this anion is similar to the VDEs predicted for (CH_3_MgCl)^−^ and (C_2_H_5_MgCl)^−^ anions yet smaller (by ca. 0.1–0.8 eV) than those calculated for the (MgX_2_)^−^ (X = F, Cl, Br) systems (cf. Tables 1–4). The relatively large vertical and adiabatic electronic stability of the (C_6_H_5_MgC_6_H_5_)^−^ anion clearly relates to the presence of two electronegative phenyl groups in this system as indicated by the comparison of its EA and VDE values to those obtained for either (CH_3_MgCH_3_)^−^ or (C_2_H_5_MgC_2_H_5_)^−^ species (Tables 2 and 3).

## 4 Conclusions

On the basis of the QCISD/aug-cc-pVDZ calculations performed for (i) representative Grignard compounds RMgX (X = F, Cl, Br; R = CH_3_, C_2_H_5_, C_6_H_5_) and their corresponding anions, (ii) magnesium halides MgX_2_ and the (MgX_2_)^−^ anions (X = F, Cl, Br), and (iii) the neutral and negatively charged RMgR molecules (R = CH_3_, C_2_H_5_, C_6_H_5_), we arrive at the following conclusions:

(i) Grignard compounds RMgX (X = F, Cl, Br) containing methyl, ethyl, or phenyl functional groups are capable of forming stable anionic states whose vertical electron detachment energies were determined to span the 0.79–1.22 eV range (for (CH_3_MgX)^−^), the 0.83–1.25 eV range (for (C_2_H_5_MgX)^−^), and the 1.24–1.62 eV range (for (C_6_H_5_MgX)^−^).
(ii) The excess electron density distribution in the RMgX^–^ anions is consistent with the umpolung character (C^δ−^ ← Mg^δ+^) of the C–Mg bond as the excess electron is localized mainly in the valence region of the Mg atom.
(iii) The adiabatic electron affinities of the RMgX (X = F, Cl, Br) neutral molecules were predicted to span the 0.26–0.54 eV range (for CH_3_MgX), the 0.30–0.58 eV range (for C_2_H_5_MgX), and the 0.59–0.86 eV range (for C_6_H_5_MgX).
(iv) The geometry relaxation of the RMgX (X = F, Cl, Br) systems upon attachment of an excess electron is substantial and relates primarily to the bending of the C–Mg–X fragment (whose alignment is linear in the neutral species).
(v) The replacement of the halogen atom in the (RMgX)^−^ anions with a methyl, ethyl, or phenyl group leads to a decrease in the excess electron binding energy, yet the resulting (RMgR)^−^ anions (R = CH_3_, C_2_H_5_, C_6_H_5_) remain both adiabatically and vertically electronically stable (EA = 0.01–0.56 eV, VDE = 0.46–1.16 eV).
(vi) The (CH_3_MgX)^−^, (C_2_H_5_MgX)^−^, and (C_6_H_5_MgX)^−^ systems were identified as valence-bound anions having their excess electron binding energies smaller than those for the anions formed by their corresponding magnesium halides (EA = 0.66–1.17 eV, VDE = 1.29–1.97 eV) whose valence-bound nature was likewise recognized.
(vii) The valence-bound nature of the (CH_3_MgX)^−^, (C_2_H_5_MgX)^−^, and (C_6_H_5_MgX)^−^ (X = F, Cl, Br) anions indicates that these species might also be stable in solution, which would not be possible if these were dipole-bound anionic states.

## Supporting Information Available

Cartesian coordinates of the structures corresponding to global and local minima (PDF)
