# Supplementary material for: An Excess Electron Bound to Magnesium Halides and Basic Grignard Compounds (RMgX and RMgR, R = Me, Et, Ph; X = F, Cl, Br)
Source: J Phys Chem A. 2021 Mar 10;125(11):2334–43. doi: 10.1021/acs.jpca.1c00750 (PMC8041300; doi:10.1021/acs.jpca.1c00750)
Supplement: Supplementary file 1 — jp1c00750_si_001.pdf [file jp1c00750_si_001.pdf]

Supporting Information

An Excess Electron Bound to Magnesium Halides and Basic Grignard Compounds (RMgX and RMgR, R=Me, Et, Ph; X=F, Cl, Br)

Jakub Brzeski[a], Sylwia Freza[a], Marcin Czapla[a], Piotr Skurski[a,b,*]

[a] *Laboratory of Quantum Chemistry, Faculty of Chemistry, University of Gdańsk, Wita Stwosza 63, 80-308 Gdańsk, Poland*

[b] *Henry Eyring Center for Theoretical Chemistry, Department of Chemistry, University of Utah, Salt Lake City, Utah 84112, United States*

[*] corresponding author: piotr.skurski@ug.edu.pl

Table S1. Cartesian coordinates (in Å) of the magnesium halides with general formula MgX_2 (X=F, Cl, Br) and their corresponding anions.

Lewis acids				
**Species**	**Coordinates**			
MgF_2	12	0.000000000	0.000000000	0.000000000
	9	0.000000000	0.000000000	1.768453000
	9	0.000000000	0.000000000	-1.768453000
$(MgF_2)^-$	12	0.000000000	0.000000000	0.515913000
	9	0.000000000	1.622112000	-0.343942000
	9	0.000000000	-1.622112000	-0.343942000
$MgCl_2$	12	0.766707000	0.000000000	0.542144000
	17	-0.509239000	0.000000000	2.346604000
	17	2.042653000	0.000000000	-1.262316000
$(MgCl_2)^-$	12	0.000000000	0.000000000	0.883677000
	17	0.000000000	2.027959000	-0.311886000
	17	0.000000000	-2.027959000	-0.311886000
$MgBr_2$	12	0.000000000	0.000000000	0.000000000
	35	0.000000000	0.000000000	2.355742000
	35	0.000000000	0.000000000	-2.355742000
$(MgBr_2)^-$	12	0.131296000	0.000000000	0.092840000
	35	-0.064484000	0.000000000	2.607733000
	35	2.437100000	0.000000000	-0.930040000

Table S2. Cartesian coordinates (in Å) of the compounds with general formula MeMgX (X=F, Cl, Br) and their corresponding anions.

Lewis acids				
**Species**	**Coordinates**			
CH_3MgF	6	0.000000000	0.000000000	-1.967841000
	1	0.000000000	1.028606000	-2.377071000
	1	-0.890799000	-0.514303000	-2.377071000
	1	0.890799000	-0.514303000	-2.377071000
	12	0.000000000	0.000000000	0.139374000
	9	0.000000000	0.000000000	1.918419000
$(CH_3MgF)^-$	6	-1.671197000	-0.819099000	0.000000000
	1	-1.644007000	-1.484892000	0.892832000
	1	-1.644007000	-1.484892000	-0.892832000
	1	-2.662449000	-0.317470000	0.000000000
	12	0.000000000	0.594731000	0.000000000
	9	1.775294000	0.118342000	0.000000000
CH_3MgCl	6	0.000000000	0.000000000	-2.570155000
	1	0.000000000	1.029204000	-2.977497000
	1	-0.891317000	-0.514602000	-2.977497000
	1	0.891317000	-0.514602000	-2.977497000
	12	0.000000000	0.000000000	-0.469086000
	17	0.000000000	0.000000000	1.763674000
$(CH_3MgCl)^-$	6	2.182621000	0.865906000	0.000000000
	1	2.572479000	0.325172000	0.891942000
	1	2.650189000	1.873729000	0.000000000
	1	2.572479000	0.325172000	-0.891942000

	12	0.000000000	0.924529000	0.000000000
	17	-1.228875000	-1.106697000	0.000000000
CH_3MgBr	6	0.000000000	0.000000000	-3.226705000
	1	0.000000000	1.029168000	-3.634242000
	1	-0.891286000	-0.514584000	-3.634242000
	1	0.891286000	-0.514584000	-3.634242000
	12	0.000000000	0.000000000	-1.126891000
	35	0.000000000	0.000000000	1.251018000
$(CH_3MgBr)^-$	6	-0.846418000	-2.797437000	0.000000000
	1	-1.505731000	-2.699451000	0.891730000
	1	-0.449540000	-3.835171000	0.000000000
	1	-1.505731000	-2.699451000	-0.891730000
	12	0.711626000	-1.270066000	0.000000000
	35	0.000000000	1.178842000	0.000000000
CH_3MgCH_3	12	0.000000000	0.000000000	0.000000000
	6	0.000000000	0.000000000	2.120318000
	6	0.000000000	0.000000000	-2.120318000
	1	0.000000000	-1.027276000	-2.538253000
	1	-0.889647000	0.513638000	-2.538253000
	1	0.889647000	0.513638000	-2.538253000
	1	0.000000000	1.027276000	2.538253000
	1	-0.889647000	-0.513638000	2.538253000
	1	0.889647000	-0.513638000	2.538253000
$(CH_3MgCH_3)^-$	6	0.000000000	1.964514000	-0.344753000
	1	-0.891945000	2.094485000	-1.000901000
	1	0.891945000	2.094485000	-1.000901000
	1	0.000000000	2.816442000	0.367370000
	12	0.000000000	0.000000000	0.617158000
	6	0.000000000	-1.964514000	-0.344753000
	1	0.000000000	-2.816442000	0.367370000
	1	-0.891945000	-2.094485000	-1.000901000
	1	0.891945000	-2.094485000	-1.000901000

Table S3. Cartesian coordinates (in Å) of the compounds with general formula EtMgX (X=F, Cl, Br) and their corresponding anions.

**Lewis acids**				
**Species**	**Coordinates**			
C_2H_5MgF	12	0.000000000	0.795124000	0.000000000
	6	-0.887991000	-1.137397000	0.000000000
	1	-1.557936000	-1.213720000	0.880955000
	1	-1.557936000	-1.213720000	-0.880955000
	6	0.097987000	-2.335484000	0.000000000
	1	0.756309000	-2.326430000	0.888482000
	1	0.756309000	-2.326430000	-0.888482000
	1	-0.428569000	-3.309231000	0.000000000
	9	0.752428000	2.409481000	0.000000000
anti-$(C_2H_5MgF)^-$	12	0.000000000	0.958481000	0.000000000
	6	0.104023000	-1.244726000	0.000000000
	1	-0.471574000	-1.605249000	0.884406000

	1 -0.471574000 -1.605249000 -0.884406000<br>6 1.500812000 -1.912662000 0.000000000<br>1 2.093409000 -1.606023000 0.885008000<br>1 2.093409000 -1.606023000 -0.885008000<br>1 1.475502000 -3.025785000 0.000000000<br>9 -1.594243000 1.876765000 0.000000000
syn-(C_2H_5MgF)$^-$	12 0.000000000 1.168165000 0.000000000<br>6 -1.317749000 -0.598820000 0.000000000<br>1 -1.995727000 -0.545990000 0.882645000<br>1 -1.995727000 -0.545990000 -0.882645000<br>6 -0.584393000 -1.965771000 0.000000000<br>1 0.074082000 -2.069542000 0.884892000<br>1 0.074082000 -2.069542000 -0.884892000<br>1 -1.262823000 -2.849185000 0.000000000<br>9 1.835441000 1.049980000 0.000000000
C_2H_5MgCl	12 0.000000000 -0.298010000 0.000000000<br>6 1.951120000 0.529656000 0.000000000<br>1 2.497423000 0.135194000 0.881221000<br>1 2.497423000 0.135194000 -0.881221000<br>6 2.026173000 2.079264000 0.000000000<br>1 1.532934000 2.514897000 0.888582000<br>1 1.532934000 2.514897000 -0.888582000<br>1 3.070718000 2.445121000 0.000000000<br>17 -2.058541000 -1.166041000 0.000000000
anti-(C_2H_5MgCl)$^-$	12 0.000000000 0.764852000 0.000000000<br>6 1.463011000 -0.874682000 0.000000000<br>1 1.242421000 -1.516917000 0.883784000<br>1 1.242421000 -1.516917000 -0.883784000<br>6 2.967370000 -0.509415000 0.000000000<br>1 3.233545000 0.101972000 0.885365000<br>1 3.233545000 0.101972000 -0.885365000<br>1 3.650439000 -1.388352000 0.000000000<br>17 -2.304980000 0.196742000 0.000000000
syn-(C_2H_5MgCl)$^-$	12 0.000000000 1.193930000 0.000000000<br>6 -1.940694000 0.163608000 0.000000000<br>1 -2.531737000 0.498344000 0.883106000<br>1 -2.531737000 0.498344000 -0.883106000<br>6 -1.848739000 -1.384074000 0.000000000<br>1 -1.292934000 -1.753779000 0.883492000<br>1 -1.292934000 -1.753779000 -0.883492000<br>1 -2.836229000 -1.898775000 0.000000000<br>17 1.954245000 -0.152630000 0.000000000
C_2H_5MgBr	12 0.000000000 0.655658000 0.000000000<br>6 -0.646558000 2.673472000 0.000000000<br>1 -0.204373000 3.181707000 0.881403000<br>1 -0.204373000 3.181707000 -0.881403000<br>6 -2.183073000 2.887060000 0.000000000<br>1 -2.661087000 2.434654000 0.888596000<br>1 -2.661087000 2.434654000 -0.888596000

	1	-2.454396000	3.960098000	0.000000000
	35	0.718946000	-1.612112000	0.000000000
anti-$(C_2H_5MgBr)^-$	12	0.000000000	1.060442000	0.000000000
	6	2.188799000	0.885310000	0.000000000
	1	2.471759000	0.268294000	0.883695000
	1	2.471759000	0.268294000	-0.883695000
	6	3.024272000	2.188579000	0.000000000
	1	2.794804000	2.814489000	0.885507000
	1	2.794804000	2.814489000	-0.885507000
	1	4.125279000	2.024777000	0.000000000
	35	-1.312481000	-1.124542000	0.000000000
syn-$(C_2H_5MgBr)^-$	12	0.576480000	-1.334757000	-0.074287000
	6	2.529130000	-0.348403000	0.114609000
	1	2.944540000	-0.587028000	1.121132000
	1	3.253557000	-0.788309000	-0.607337000
	6	2.484922000	1.190546000	-0.066817000
	1	1.769884000	1.657105000	0.637274000
	1	2.134227000	1.465489000	-1.081113000
	1	3.467023000	1.693898000	0.080986000
	35	-1.444894000	0.214945000	0.012964000
$C_2H_5MgC_2H_5$	12	0.000000000	0.000000000	0.440450000
	6	0.000000000	2.138238000	0.422494000
	1	-0.320115000	2.510086000	1.419063000
	1	1.044890000	2.497751000	0.308796000
	6	-0.881772000	2.802959000	-0.669291000
	1	-1.945988000	2.516853000	-0.568188000
	1	-0.567414000	2.506092000	-1.687959000
	1	-0.844195000	3.909387000	-0.633629000
	6	0.000000000	-2.138238000	0.422494000
	1	0.320115000	-2.510086000	1.419063000
	1	-1.044890000	-2.497751000	0.308796000
	6	0.881772000	-2.802959000	-0.669291000
	1	1.945988000	-2.516853000	-0.568188000
	1	0.567414000	-2.506092000	-1.687959000
	1	0.844195000	-3.909387000	-0.633629000
anti-$(C_2H_5MgC_2H_5)^-$	12	0.000000000	0.000000000	0.499990000
	6	0.000000000	1.931269000	-0.564533000
	1	0.883230000	1.926433000	-1.247224000
	1	-0.883230000	1.926433000	-1.247224000
	6	0.000000000	3.253367000	0.242360000
	1	0.885006000	3.318289000	0.906185000
	1	-0.885006000	3.318289000	0.906185000
	1	0.000000000	4.173502000	-0.384824000
	6	0.000000000	-1.931269000	-0.564533000
	1	-0.883230000	-1.926433000	-1.247224000
	1	0.883230000	-1.926433000	-1.247224000
	6	0.000000000	-3.253367000	0.242360000
	1	-0.885006000	-3.318289000	0.906185000
	1	0.885006000	-3.318289000	0.906185000

	1 0.000000000 -4.173502000 -0.384824000
syn-$(C_2H_5MgC_2H_5)^-$	12 0.000000000 1.019810000 0.000000000<br>6 -2.153446000 0.541660000 0.000000000<br>1 -2.653653000 1.001411000 0.882757000<br>1 -2.653653000 1.001411000 -0.882757000<br>6 -2.428736000 -0.986473000 0.000000000<br>1 -1.977262000 -1.477789000 0.885238000<br>1 -1.977262000 -1.477789000 -0.885238000<br>1 -3.508168000 -1.261404000 0.000000000<br>6 1.525733000 -0.571756000 0.000000000<br>1 1.313429000 -1.221508000 0.882544000<br>1 1.313429000 -1.221508000 -0.882544000<br>6 3.029854000 -0.203383000 0.000000000<br>1 3.292935000 0.409497000 0.884995000<br>1 3.292935000 0.409497000 -0.884995000<br>1 3.716836000 -1.079828000 0.000000000

Table S4. Cartesian coordinates (in Å) of the compounds with general formula PhMgX (X=F, Cl, Br).

Lewis acids	
**Species**	**Coordinates**
C_6H_5MgF	6 0.032485000 0.000000000 0.000000000<br>6 -0.722169000 -1.206891000 0.000000000<br>6 -2.131276000 -1.215204000 0.000000000<br>6 -2.839734000 0.000001000 0.000000000<br>6 -2.131275000 1.215204000 0.000000000<br>6 -0.722168000 1.206891000 0.000000000<br>1 -0.209699000 -2.177975000 0.000000000<br>1 -2.674699000 -2.166312000 0.000000000<br>1 -3.934582000 0.000001000 0.000000000<br>1 -2.674697000 2.166314000 0.000000000<br>1 -0.209697000 2.177974000 0.000000000<br>12 2.132867000 -0.000001000 0.000000000<br>9 3.910420000 0.000001000 0.000000000
$(C_6H_5MgF)^-$	6 0.078446000 0.162898000 0.002840000<br>6 -0.497265000 -1.141429000 -0.048607000<br>6 -1.889916000 -1.373040000 -0.059919000<br>6 -2.784203000 -0.285417000 -0.019337000<br>6 -2.265130000 1.022677000 0.032160000<br>6 -0.868064000 1.226276000 0.042395000<br>1 0.168604000 -2.015433000 -0.081308000<br>1 -2.280092000 -2.398828000 -0.100274000<br>1 -3.867860000 -0.454667000 -0.027714000<br>1 -2.949674000 1.880919000 0.064219000<br>1 -0.504571000 2.263627000 0.083152000<br>12 2.267882000 0.386112000 0.015170000<br>9 3.255589000 -1.163895000 -0.043148000
C_6H_5MgCl	6 -0.000002000 0.521037000 0.000000000<br>6 1.207026000 1.275204000 0.000000000

	6	1.215458000	2.684211000	0.000000000
	6	0.000002000	3.392305000	0.000000000
	6	-1.215456000	2.684214000	0.000000000
	6	-1.207028000	1.275207000	0.000000000
	1	2.177936000	0.762407000	0.000000000
	1	2.166342000	3.227953000	0.000000000
	1	0.000003000	4.487177000	0.000000000
	1	-2.166339000	3.227958000	0.000000000
	1	-2.177938000	0.762412000	0.000000000
	12	-0.000002000	-1.573830000	0.000000000
	17	0.000001000	-3.798530000	0.000000000
$(C_6H_5MgCl)^-$	6	-0.519348000	-0.453416000	0.000000000
	6	-1.686958000	-1.268297000	0.000000000
	6	-2.997448000	-0.742958000	0.000000000
	6	-3.194490000	0.651322000	0.000000000
	6	-2.070572000	1.500553000	0.000000000
	6	-0.770734000	0.950302000	0.000000000
	1	-1.577005000	-2.362726000	0.000000000
	1	-3.864161000	-1.417256000	0.000000000
	1	-4.208289000	1.069800000	0.000000000
	1	-2.209602000	2.589696000	0.000000000
	1	0.084213000	1.640476000	0.000000000
	12	1.544027000	-1.190995000	0.000000000
	17	3.137308000	0.554324000	0.000000000
C_6H_5MgBr	6	0.000000000	0.000000000	-1.600293000
	6	-1.206549000	0.000000000	-2.354924000
	6	-1.215102000	0.000000000	-3.763807000
	6	0.000000000	0.000000000	-4.472477000
	6	1.215102000	0.000000000	-3.763807000
	6	1.206549000	0.000000000	-2.354924000
	1	-2.177607000	0.000000000	-1.842273000
	1	-2.166740000	0.000000000	-4.306145000
	1	0.000000000	0.000000000	-5.567235000
	1	2.166740000	0.000000000	-4.306145000
	1	2.177607000	0.000000000	-1.842273000
	12	0.000000000	0.000000000	0.494086000
	35	0.000000000	0.000000000	2.862149000
$(C_6H_5MgBr)^-$	6	-0.260643000	0.201246000	-1.540731000
	6	-1.013781000	0.907204000	-2.521264000
	6	-0.849641000	0.712641000	-3.909990000
	6	0.096650000	-0.217629000	-4.380865000
	6	0.866749000	-0.941292000	-3.449335000
	6	0.683981000	-0.728791000	-2.066131000
	1	-1.763453000	1.642996000	-2.194993000
	1	-1.456907000	1.283794000	-4.624679000
	1	0.232053000	-0.376379000	-5.457586000
	1	1.608185000	-1.669782000	-3.802820000
	1	1.299586000	-1.308374000	-1.364711000
	12	-0.458822000	0.448576000	0.624855000

	35	1.136170000	-1.067298000	1.882714000
$C_6H_5MgC_6H_5$	6	0.000000000	0.000000000	-2.112180000
	6	-0.852726000	-0.852726000	-2.871474000
	6	-0.859506000	-0.859506000	-4.281610000
	6	0.000000000	0.000000000	-4.990728000
	6	0.859506000	0.859506000	-4.281610000
	6	0.852726000	0.852726000	-2.871474000
	1	-1.540157000	-1.540157000	-2.360685000
	1	-1.532086000	-1.532086000	-4.824751000
	1	0.000000000	0.000000000	-6.085197000
	1	1.532086000	1.532086000	-4.824751000
	1	1.540157000	1.540157000	-2.360685000
	12	0.000000000	0.000000000	0.000002000
	6	0.000000000	0.000000000	2.112180000
	6	0.852727000	-0.852727000	2.871474000
	6	0.859509000	-0.859509000	4.281611000
	6	0.000000000	0.000000000	4.990723000
	6	-0.859509000	0.859509000	4.281611000
	6	-0.852727000	0.852727000	2.871474000
	1	1.540157000	-1.540157000	2.360684000
	1	1.532087000	-1.532087000	4.824753000
	1	0.000000000	0.000000000	6.085193000
	1	-1.532087000	1.532087000	4.824753000
	1	-1.540157000	1.540157000	2.360684000
$(C_6H_5MgC_6H_5)^-$	12	0.176393000	0.000000000	0.124734000
	6	-0.016578000	0.000000000	2.322408000
	6	2.184074000	0.000000000	-0.789757000
	6	2.378560000	-0.000001000	-2.202359000
	6	3.651472000	-0.000001000	-2.816093000
	6	4.813315000	0.000000000	-2.019551000
	6	4.680909000	0.000001000	-0.617204000
	6	3.393807000	0.000001000	-0.033288000
	1	1.498417000	-0.000002000	-2.861259000
	1	3.738876000	-0.000002000	-3.910149000
	1	5.806311000	0.000000000	-2.484158000
	1	5.576599000	0.000002000	0.016632000
	1	3.340885000	0.000002000	1.063227000
	6	-1.283572000	0.000001000	2.976641000
	6	-1.437874000	0.000001000	4.381338000
	6	-0.299612000	0.000000000	5.211225000
	6	0.978395000	-0.000001000	4.618929000
	6	1.099876000	-0.000001000	3.210799000
	1	-2.198177000	0.000002000	2.366477000
	1	-2.440221000	0.000002000	4.828437000
	1	-0.406645000	0.000000000	6.302299000
	1	1.874550000	-0.000002000	5.252108000
	1	2.116035000	-0.000002000	2.795386000
